# Department of Clinical Pathology

**Published:** 1916-11

**Authors:** 

**Affiliations:** 203 Hicks Bldg., San Antonio, Texas


### Department of Clinical Pathology

Dr. B. F. Stout, Editor, 203 Hicks Bldg., San Antonio, Texas

Communications solicited and should be sent direct to Dr. Stout. Copy should be in Dr. Stout's possession by the 20th of the preceding month.

In taking up the work of this department it seems best to state briefly the purpose of it as understood by the writer. While a knowledge of pathology and bacteriology and its applications to every-day practice is absolutely necessary to proper diagnosis and treatment, the average physician is unable to keep up with the special literature on the subject. No one man who is busy with his routine professional work can possibly find the time to read enough to keep up with all the departments of medicine. It, therefore, must be presented in such a condensed form that the more important points may be available for the busy man. The purpose of this department will be to review the more important advances with such comments as may be explanatory and helpful. Every effort will be made to make the subject clear and as brief as is consistent with clarity.

In view of the widespread interest in epidemic poliomyelitis just at this time it will not be amiss to call attention to the reports of the researches conducted during the recent epidemic and published since in the *Jr. A. M. A.* by Rosenow and his associates working in New York, and Nuzum and Herzog in Chicago. Those interested enough can read the original articles for details. The astonishing thing about it seems to be that they have found a streptococcus, which exists in different forms, one small enough to pass a Berkefeld filter, and a larger form readily demonstrated by ordinary stains. The work has been very carefully done, and while these investigators do not go so far as to claim this organism as the cause of the disease, it would seem very likely that it is so.

In 1909 Flexner and Lewis demonstrated that the virus would pass through porcelain filters, and cultures were obtained later (1913) by Noguchi and Flexner. They apparently obtained both forms of the organism, but regarded the large form as a contamination.

Mathers published a short report in September of this year describing the same streptococcus.

Rosenow, approaching the problem with his newer methods of tissue cultures and without being discouraged by the ultra-microscopic nature of the virus, has apparently demonstrated that the organism may be made to assume both the ultra-microscopic size or the usual size at will according to the culture method employed. The large form has been grown from the filtrate and the disease produced in animals. The streptococcus has an elective affinity for nerve tissues, as have the various other streptococci described by Rosenow, respectively, affinities for endocardium, joints, muscles, ovarian tissue, gall, bladder, etc. The organism has been isolated from tonsils and throats of infected individuals, throwing light on the manner of its spread from infected patients and healthy carriers. In respect to the variability of size it must be recalled that the virus of rabies is a filterable one, that is, it passes through porcelain filters that hold back the ordinary bacteria—while in the brains of infected animals the organism appears as the Negri bodies—large enough to be readily recognized by the ordinary microscopic magnification.

This work carried farther will open up new fields for the investigation of the other filterable viruses—of which yellow fever is the most important.

Krause and later Foster have demonstrated that acute colds are in some epidemics caused by organisms of the ultra-microscopic type—the virus passing through Berkefeld N filters. In view of the fact that the secretions from the nose in early rhinitis is nearly always sterile while later pure cultures of members of the streptococcus groups appear, the writer considers it quite possible that an organism of the polymorphous type of streptococcus may be the cause of epidemic colds. That the streptococcus is an etiological factor in colds, the writer considers proven from the fact of its constant presence in the nasal secretions after the first few days and by the very marked improvement in these cases by the use of vaccines. Investigation of this organism by Rosenow's methods of culture would doubtless clear up this point. In view of the undoubted mutation between the members of the streptococcus-pneumococcus group it is quite conceivable that the cold organism might attain an elective affinity for central nerve tissues and thus become the cause of poliomyelitis. One might go on indefinitely speculating on the fields opened up by this brilliant work. It is certainly not too much to say that this whole problem of prevention and cure of poliomyelitis may be cleared up in a year's time.

B. F. STOUT.

